# Relationship Between Plantar Tissue Hardness and Plantar Pressure Distributions in People With Diabetic Peripheral Neuropathy

**DOI:** 10.3389/fbioe.2022.836018

**Published:** 2022-04-04

**Authors:** Yijie Duan, Weiyan Ren, Wei Liu, Jianchao Li, Fang Pu, Yih-Kuen Jan

**Affiliations:** ^1^ Key Laboratory of Biomechanics and Mechanobiology, Ministry of Education, Beijing Advanced Innovation Center for Biomedical Engineering, School of Biological Science and Medical Engineering, Beihang University, Beijing, China; ^2^ Key Laboratory of Rehabilitation Technical Aids for Old-Age Disability, Key Laboratory of Human Motion Analysis and Rehabilitation Technology of the Ministry of Civil Affairs, National Research Center for Rehabilitation Technical Aids, Beijing, China; ^3^ Department of Kinesiology and Community Health, University of Illinois at Urbana-Champaign, Champaign, IL, United States

**Keywords:** diabetic peripheral neuropathy, foot ulcers, plantar soft tissue hardness, plantar pressure, plantar loading

## Abstract

**Objective:** People with diabetic peripheral neuropathy (DPN) are usually accompanied with increased plantar pressure. Such high plantar loading during daily activities may cause changes in the biomechanical properties of plantar soft tissue, whose viability is critical to the development of foot ulcers. This study aimed to investigate the relationship between plantar tissue hardness and plantar pressure in people with and without DPN, and preliminarily explore the influence of plantar loading patterns on the plantar pressure and tissue hardness.

**Methods:** The study was conducted on 14 people with DPN and 14 diabetic people without DPN. The Shore durometer and MatScan System were used to measure the plantar tissue hardness and plantar pressure, respectively. The plantar loading level was evaluated by the duration of daily weight-bearing activity and was used to group diabetic participants with and without DPN into two subgroups (lower loading group and higher loading group).

**Results:** The plantar tissue hardness was significantly correlated with static peak plantar pressure (PPP, *p* < 0.05) and dynamic pressure-time integral (PTI, *p* < 0.05) in the forefoot region in people with DPN. Results of variance analysis showed a significant interaction effect between peripheral neuropathy and plantar loading on tissue hardness (*p* < 0.05), but not plantar pressure. For people with DPN, significant differences in tissue hardness between the higher loading group and lower loading group were observed in the forefoot, midfoot and hindfoot regions. In the higher loading group, people with DPN had significantly greater tissue hardness than that in people without DPN in the toes, forefoot, midfoot and hindfoot regions (*p* < 0.05).

**Conclusions:** There is a significant correlation between tissue hardness and PPP, and between tissue hardness and PTI in people with DPN. Plantar loading associated with daily activities plays a significant role on the plantar tissue hardness in people with DPN. The findings of this study contribute to further understand the relationship between increased plantar tissue hardness and high plantar pressure in people with diabetic peripheral neuropathy.

## Introduction

Diabetic foot ulcers (DFUs) are one of the most serious complications of diabetes, with a global prevalence of 6.3% (Zhang et al., 2017). Studies have shown that the amputation rate in diabetics is much higher than non-diabetics (Ahmad et al., 2016; Claessen et al., 2018; Gurney et al., 2018), which seriously affects the physical health and imposes additional financial burden for people with diabetes.

Peripheral neuropathy is an important risk factor for DFUs (Monteiro-Soares et al., 2012; Armstrong et al., 2017), which may lead to foot deformities, biomechanical abnormalities, and the loss of protective sensation (Volmer-Thole and Lobmann, 2016). Several studies demonstrated that people with diabetic peripheral neuropathy (DPN) have a higher peak plantar pressure (Sacco et al., 2014; Halawa et al., 2018) and show an imbalance in plantar pressure distribution (Caselli et al., 2002; Kernozek et al., 2013; Al-Angari et al., 2017), compared with people without DPN. The loss of protective sensation caused by neuropathy also prevents people with DPN from responding promptly to abnormal mechanical stress during daily activities (Armstrong et al., 2017). These factors may affect their ambulatory function.

Plantar soft tissue is the first contact with the ground during daily activities, such as standing and walking, and plays a key role in shock-absorbing and protecting foot from external mechanical damage. The accumulation of advanced glycation end-products in people with diabetes can lead to histological changes in plantar soft tissue (Ramasamy et al., 2005). Abnormal microvascular function and dysfunctional secretion of sweat caused by peripheral neuropathy may further aggravate histological changes (Volmer-Thole and Lobmann, 2016). Several studies have found that plantar soft tissue of people with DPN was thinner and stiffer than healthy people (Klaesner et al., 2002; Chao et al., 2011; Sun et al., 2011; Jan et al., 2013). Periyasamy et al. further reported significant differences in plantar tissue hardness between diabetic people with and without DPN using a shore durometer (Periyasamy et al., 2012a; Periyasamy et al., 2012b). Increased tissue hardness in people with DPN may be accompanied by stress concentration during daily activities (Klaesner et al., 2002; Chatzistergos et al., 2014). Over time, the dry skin and abnormal plantar pressure may cause hyperkeratosis under repeated and elevated plantar pressure loading (Volmer-Thole and Lobmann, 2016), which may affect the biomechanical properties of the soft tissue, and increase the vulnerability of plantar tissue to trauma and ulceration.

Several studies have shown the potential link between plantar pressure and the biomechanical properties of soft tissue (Gefen, 2003; Jan et al., 2013; Helili et al., 2021). Jan et al.'s study demonstrated a correlation between the soft tissue biomechanical properties and plantar pressure gradient in the first metatarsal region in people with DPN (Jan et al., 2013). Helili et al. also reported a correlation between plantar soft tissue hardness and average dynamic pressure in healthy people (Helili et al., 2021). However, the whole plantar regions and more plantar pressure characteristics (both peak plantar pressure and pressure-time integral (Duckworth et al., 1985; Patry et al., 2013; Chatwin et al., 2020) in static and dynamic conditions) need to be considered, in order to better understand the relationship between the biomechanical properties of soft tissue and plantar pressure distribution in people with DPN.

In daily activities, plantar loading may be an important factor affecting the plantar pressure and tissue hardness of people with DPN. Limited joint mobility, muscular alterations and foot deformities associated with peripheral neuropathy may altered the postural control and balance function during gait (Simoneau et al., 1994; Periyasamy et al., 2012a; Volmer-Thole and Lobmann, 2016), which results in an insecure gait. Such deficit of balance and posture may exacerbate their plantar biomechanical abnormalities under the excessive mechanical stress stimulus. In addition, studies showed that different levels of plantar loading have an effect on microvascular regulation (Wu et al., 2020; Duan et al., 2021). Excessive plantar pressure load may increase the degree of compression of plantar tissue and the occlusion duration of microvessels, which leads to an insufficient blood perfusion and a lack of nutrients in soft tissue. These factors may jointly influence the biomechanical properties of plantar soft tissue. It is of great significance to explore the effects of plantar loading on plantar pressure and tissue hardness for understanding the changes of soft tissue biomechanical properties on the development of ulceration in people with DPN.

Therefore, this study aimed to investigate the relationship between plantar tissue hardness and plantar pressure in people with and without DPN, and preliminarily explore the influence of plantar loading on plantar pressure and tissue hardness. This study hypothesized that the increased plantar tissue hardness was related to plantar pressure, and the plantar loading associated with daily activities has an effect on the plantar pressure and tissue hardness.

## Materials and Methods

### Participants

People with type 2 diabetes were recruited from nearby hospitals and communities. The inclusion criteria were: 1) diagnosed type 2 diabetes mellitus, 2) no symptoms such as redness, inflammation, or wounds on the skin of the feet or legs, 3) no history of amputation, and 4) performed regular moderate-intensity physical activities at least 150 min/week over the course of one year on the basis of self-report (American Diabetes Association, 2021). Moderate-intensity physical activity was defined as a metabolic equivalent (MET) of 3–5.9 Mets, according to the compendium of physical activities (Ainsworth et al., 2011).

The 10 g Semmes-Weinstein monofilament was used to identify peripheral neuropathy in people with diabetes. Participants who were unable to sense the touch of the 10 g monofilament at all four areas on the plantar surface (1st, 3rd and 5th metatarsal heads and distal hallux) were assigned to the diabetic peripheral neuropathy group (DPN group) (Boulton et al., 2008), otherwise, the participant was assigned to the non-diabetic peripheral neuropathy group (Non-DPN group).

The criterion of physical activities is the minimum weekly physical activity level recommended by ADA guidelines for people with diabetes (American Diabetes Association, 2021). The type, frequency and duration of daily physical activities of each participant were firstly recorded using the International Physical Activity Questionnaire (IPAQ) (Mynarski et al., 2012), which has been proven to be a validated tool for physical activity assessment. The median duration of weight-bearing physical activity per day of all participants (LeMaster et al., 2003) was used to divided participants of each group into two subgroups (lower loading group and higher loading group).

This study was conducted in accordance with clinical protocols approved by the institutional review board of Affiliated Hospital of National Research Center for Rehabilitation Technical Aids (20190101) and the Declaration of Helsinki (2013 revision). All participants were briefed on the study purposes and procedures and gave written informed consent prior to participation.

### Measurement of Plantar Tissue Hardness and Plantar Pressure

All tests were performed in a climate-controlled room at 24°C.

**FIGURE 1:**
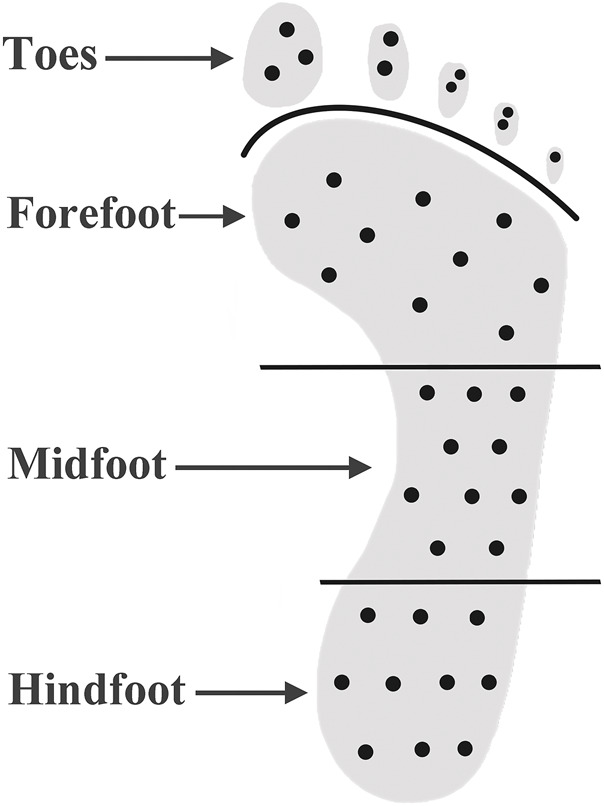
Measurement of plantar soft tissue hardness by using the Shore (OO) durometer. The black dots indicate the measurement site of plantar tissue hardness.

A Shore durometer (Model 1600, Type OO, Rex Co., Buffalo Grove, USA) was used to measure the plantar tissue hardness, which has been used in several studies (Periyasamy et al., 2012b; Helili et al., 2021). During measurement, the durometer was pressed perpendicular to the plantar skin surface and expresses the hardness in degrees of Shore (unit: °Shore). A lower Shore value indicates a softer material. The plantar surface was divided into four regions: toes, forefoot, midfoot and hindfoot. Ten sites were selected for each of four regions of interest. Plantar tissue hardness over these sites were measured and mean values of tissue hardness were calculated for comparisons to investigate the relationship between plantar tissue hardness and plantar pressure. All measurements were performed by one skilled experimenter. Figure 1 shows the measurement sites of plantar tissue hardness by using the Shore (OO) durometer. The locations and area of callus were special recorded.

A MatScan System (HR Mat, Tekscan, Inc., Boston, USA) was used to measure plantar pressure. It has a spatial resolution of 4 Sensels™/cm^2^ (25 Sensels/in^2^) with 8,448 individual pressure sensing locations. After calibration based on the manufacturer recommendations, the pressure was recorded at static conditions (standing) and dynamic conditions (taking one step on the MatScan) (Sacco et al., 2014). Recordings were made at 50 Hz for 30 s, and the analysis was made using the FootMat Research software. Plantar pressure parameters included static peak plantar pressure (PPP), dynamic peak plantar pressure (PPP) and dynamic pressure-time integral (PTI).

### Data and Statistical Analyses

The plantar foot was divided into four regions, including toes, forefoot, midfoot, and hindfoot. The plantar tissue hardness of the whole foot was the average value of the tissue hardness in the regions of toes, forefoot, midfoot and hindfoot. Static PPP, dynamic PPP and PTI of corresponding area (toes, forefoot, midfoot, hindfoot, and whole foot) were calculated to assess plantar pressure distribution. The average values of tissue hardness and plantar pressure in the corresponding regions of the left and right feet were calculated and compared.

The correlations between tissue hardness and plantar pressure in each plantar region were determined using Pearson correlation analysis. When taking the plantar loading into consideration, two-way analysis of variance (ANOVA) was used to compare the plantar tissue hardness and plantar pressure between four subgroups to investigate the effect of plantar loading and neuropathy on tissue hardness and plantar pressure in people with diabetes. If there was a significant interaction between neuropathy and plantar loading, the simple effect (examined through univariate ANOVA) was used to assess the effect of neuropathy with restricted levels of plantar loading and vice versa. If no interaction was found, the main effects of neuropathy and plantar loading on tissue hardness and plantar pressure were assessed, respectively. The main effect is defined as an integrated effect of neuropathy, which disregard the levels of plantar loading, and vice versa.

The significant level was set as 0.05. All statistical analyses were performed in SPSS (Version 26.0, IBM, Armonk, NY, USA).

## Results

A total of 28 people with diabetes volunteered in this study, including 14 people with DPN (DPN group) and 14 people without DPN (Non-DPN group). Participants’ characteristics are shown in Table 1. The median duration of daily weight-bearing activities of all participants was 2 h per day. In the DPN group, nine participants were divided into the higher loading group, and five participants were divided into the lower loading group. In the Non-DPN group, eight participants were divided into the higher loading group, and six participants were divided into the lower loading group. Except for one participant in the Non-DPN group engaged in square dancing and walking, the other participants only performed walking during daily activities. The daily weight-bearing physical activities duration of DPN group and Non-DPN group was 1.93 ± 0.92 h/day and 1.75 ± 0.80 h/day, respectively.

**TABLE 1:** Demographic and physiological information of participants in DPN group and Non-DPN group (Mean ± SD).

Variables	DPN Group	Non-DPN Group
Gender (Male/Female)	5/9	7/7
Age (years)	67.93 ± 5.72	67.86 ± 6.20
BMI (kg/m^2^)	25.95 ± 2.77	25.91 ± 2.77
Systolic blood pressure (mmHg)	136.92 ± 11.54	132.43 ± 11.88
Diastolic blood pressure (mmHg)	71.38 ± 6.47	69.29 ± 7.39
Heart rate (bpm)	71.15 ± 7.94	73.36 ± 7.69
Duration of diabetes (years)	17.64 ± 11.88	14.82 ± 6.52
Fasting blood glucose (mmol/L)	7.90 ± 1.79	8.09 ± 1.65
ABI (left)	1.08 ± 0.12	1.03 ± 0.08
ABI (right)	1.00 ± 0.13	1.08 ± 0.06

There was no significant difference in all parameters between the DPN group and Non-DPN group (*p* > 0.05). BMI: body mass index; ABI: Ankle-brachial index. DPN: people with diabetic peripheral neuropathy; Non-DPN: people without diabetic peripheral neuropathy.

In DPN group, three participants had callus over the forefoot region, two participants had callus over the big toe, and one participant had callus over both forefoot region and big toe. In the Non-DPN group, none of them had callus in their feet.

### Relationships Between Soft Tissue Hardness and Plantar Pressure

For all participants, tissue hardness in the forefoot region was significantly correlated with static PPP and dynamic PTI (Static PPP: *r* = 0.556, *p* = 0.002, and Dynamic PTI: *r* = 0.447, *p* = 0.017). No significant correlations between tissue hardness and plantar pressure in other plantar regions were observed (*p* > 0.05).

**FIGURE 2:**
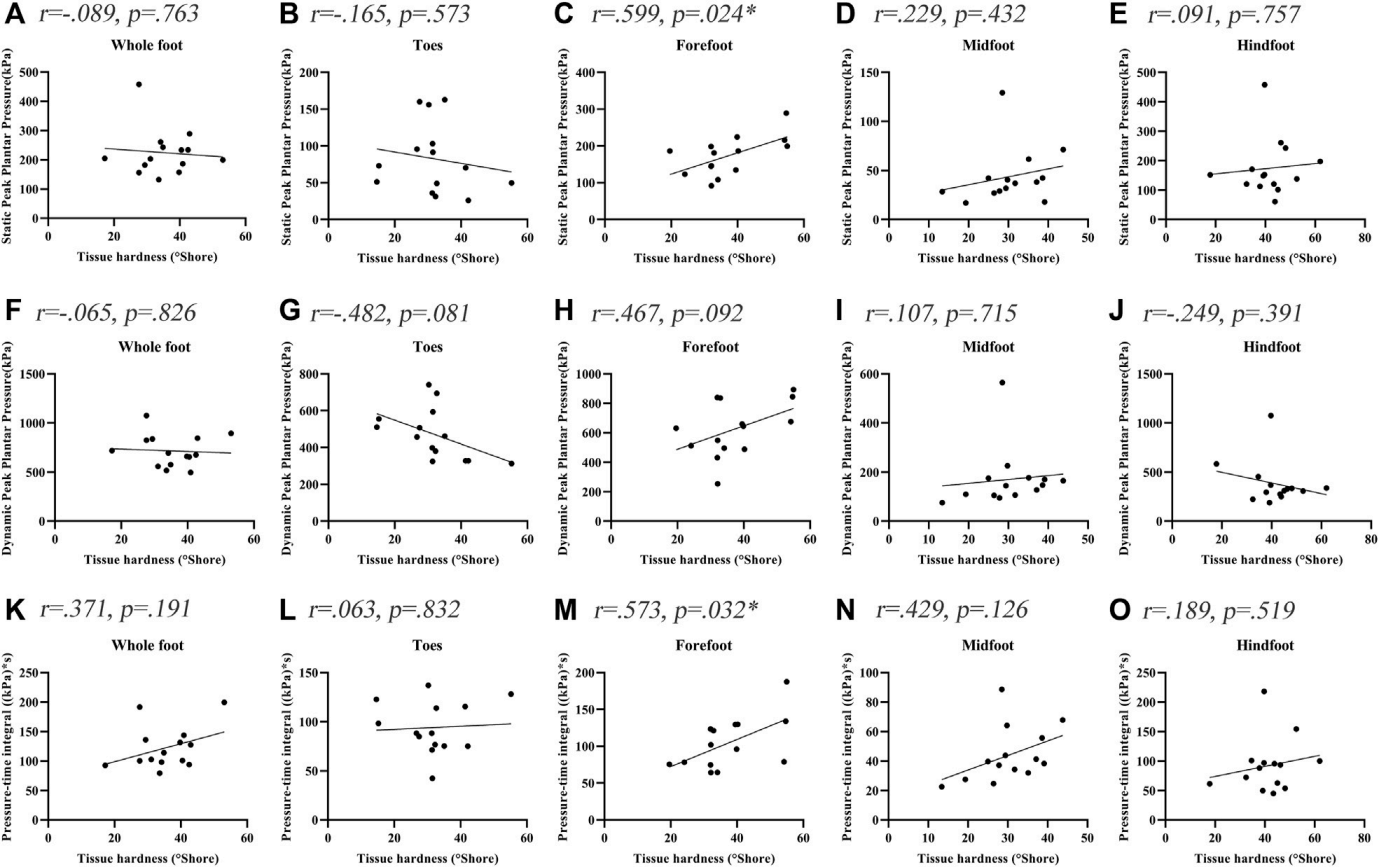
Correlation coefficients between plantar tissue hardness and plantar pressure in people with DPN. * indicates a significant correlation (*p* < 0.05). **(A-E)** represent the correlation coefficients between plantar tissue hardness and static peak plantar pressure in the whole foot, toes region, forefoot region, midfoot region and hindfoot region, respectively. **(F-J)** represent the correlation coefficients between plantar tissue hardness and dynamic peak plantar pressure in the whole foot, toes region, forefoot region, midfoot region and hindfoot region, respectively. **(K-O)** represent the correlation coefficients between plantar tissue hardness and pressure-time integral in the whole foot, toes region, forefoot region, midfoot region and hindfoot region, respectively.

Figure 2 shows the correlations between the soft tissue hardness and plantar pressure in each plantar region of people with DPN. The tissue hardness of the forefoot region was significantly correlated with static PPP and dynamic PTI (tissue hardness and static PPP: *r* = 0.599, *p* = 0.024, tissue hardness and Dynamic PTI: *r* = 0.573, *p* = 0.032). No significant correlations between the soft tissue hardness and plantar pressure in other plantar regions were observed (*p* > 0.05).

**FIGURE 3:**
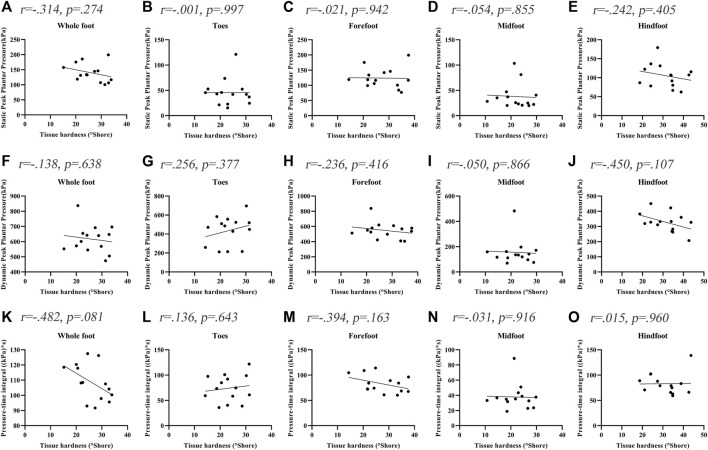
Correlation coefficients between plantar tissue hardness and plantar pressure in people without DPN. **(A‐E)** represent the correlation coefficients between plantar tissue hardness and static peak plantar pressure in the whole foot, toes region, forefoot region, midfoot region and hindfoot region, respectively. **(F‐J)** represent the correlation coefficients between plantar tissue hardness and dynamic peak plantar pressure in the whole foot, toes region, forefoot region, midfoot region and hindfoot region, respectively. **(K‐O)** represent the correlation coefficients between plantar tissue hardness and pressure-time integral in the whole foot, toes region, forefoot region, midfoot region and hindfoot region, respectively.

Figure 3 shows the correlations between the soft tissue hardness and plantar pressure in each plantar region of people without DPN. The correlations between the soft tissue hardness and plantar pressure both did not reach statistical significance in each plantar region (*p* > 0.05).

### Effect of Plantar Loading and Neuropathy on Tissue Hardness and Plantar Pressure

The interaction and main effect of peripheral neuropathy and plantar loading on tissue hardness and plantar pressure was showed in Table 2.

**TABLE 2:** The interaction and main effects of peripheral neuropathy and plantar loading patterns on plantar tissue hardness and plantar pressure (Mean ± SD).

		DPN	Non-DPN	Lower Loading	Higher Loading	ANOVA *p* Value		
						*P*_*I*_	*P*_*N*_	*P*_*L*_
Tissue hardness (°Shore)	Whole foot	35.39 ± 8.81	26.12 ± 5.72	27.24 ± 5.61	33.03 ± 9.67	**0.019**	**0.006**	**0.043**
	Toes	31.94 ± 10.29	23.44 ± 5.61	24.39 ± 6.17	29.82 ± 10.35	0.261	**0.026**	0.127
	Forefoot	37.32 ± 10.91	27.51 ± 7.24	28.25 ± 6.05	35.11 ± 11.78	**0.019**	**0.022**	0.053
	Midfoot	30.34 ± 8.14	21.94 ± 5.44	23.31 ± 6.21	27.97 ± 8.7	**0.045**	**0.009**	0.099
	Hindfoot	41.58 ± 10.19	31.5 ± 7.74	33.86 ± 8.99	38.27 ± 10.9	**0.016**	**0.018**	0.225
Static PPP (kPa)	Whole foot	224.15 ± 79.87	139.61 ± 30.56	177.22 ± 41.92	184.9 ± 89.14	0.170	**0.003**	0.918
	Toes	82.49 ± 48.01	45.68 ± 26.56	78.55 ± 49.51	54.72 ± 35.64	0.473	**0.009**	0.075
	Forefoot	173.47 ± 52.67	123.79 ± 33.64	140.16 ± 41.24	154.11 ± 55.76	0.060	**0.018**	0.497
	Midfoot	43.75 ± 28.75	37.79 ± 24.92	28.03 ± 6.77	49.01 ± 31.29	0.777	0.703	**0.049**
	Hindfoot	173.98 ± 97.63	105.15 ± 31.32	137.65 ± 51.78	140.79 ± 94.52	0.541	**0.035**	0.961
Dynamic PPP (kPa)	Whole foot	716.27 ± 162.5	616.08 ± 92.34	658.84 ± 109.2	670.92 ± 158.93	0.712	0.062	0.941
	Toes	471.09 ± 138.3	437.01 ± 153.78	547.55 ± 95.13	393.54 ± 140.38	0.568	0.306	**0.004**
	Forefoot	625.43 ± 184.92	546.46 ± 108.96	521.65 ± 151.07	627.55 ± 145.65	0.472	0.283	0.087
	Midfoot	170.56 ± 120.44	154.94 ± 100.86	107.66 ± 21.81	198.39 ± 128.16	0.809	0.865	**0.037**
	Hindfoot	380.85 ± 222.22	327.43 ± 63.78	331.2 ± 106.24	368.98 ± 192.42	0.686	0.394	0.618
Dynamic PTI (kPa*s)	Whole foot	122.24 ± 36.23	108.36 ± 12.07	107.02 ± 14.73	120.65 ± 32.49	0.243	0.326	0.217
	Toes	94.08 ± 26.41	74.09 ± 26.46	92.87 ± 20.78	78.41 ± 30.88	0.993	**0.048**	0.127
	Forefoot	104.3 ± 35.16	82.67 ± 17.69	85.16 ± 21.19	98.88 ± 33.22	0.053	0.121	0.218
	Midfoot	44.09 ± 18.72	37.77 ± 16.82	32.17 ± 7.16	46.6 ± 20.32	0.645	0.484	**0.042**
	Hindfoot	92.2 ± 46.09	82.86 ± 20.97	83.68 ± 22.44	90.02 ± 42.31	0.573	0.621	0.683

*P*_*I*_, is the interaction between plantar loading and peripheral neuropathy; *P*_*N*_, is the main effect of peripheral neuropathy; *P*_*L*_, is the main effect of plantar loading. P value in bold text indicate the significant interaction or main effect. DPN: people with diabetic peripheral neuropathy; Non-DPN: people without diabetic peripheral neuropathy.

There was an interaction between the peripheral neuropathy and plantar loading on tissue hardness, with a statistical significance over the whole foot, forefoot, midfoot, and hindfoot region (*p* < 0.05). The results of simple effect on tissue hardness showed that people with DPN and higher loading had significantly higher tissue hardness, compared with people without DPN and higher loading (*p* < 0.05, Table3). Similarly, significant differences were also found between people with DPN and higher loading and people with DPN and lower loading, and between people with DPN and higher loading people and people without DPN and lower loading (*p* < 0.05). No significant differences in tissue hardness were observed among other subgroups (*p* > 0.05).

**TABLE 3:** The effect of peripheral neuropathy and plantar loading patterns on plantar tissue hardness (Mean ± SD).

	DPN Group		Non-DPN Group		ANOVA *p* Value		
	Lower Loading	Higher Loading	Lower Loading	Higher Loading	*P*_*LH*_	*P*_*HH*_	*P*_*HL*_
Whole foot	27.92 ± 6.47	39.54 ± 7.13^a,b,c^	26.68 ± 5.35	25.7 ± 6.31	**0.011**	**0.001**	**0.002**
Toes	26.46 ± 6.72	34.98 ± 10.96^b,c^	22.67 ± 5.67	24.01 ± 5.89	0.144	**0.024**	**0.026**
Forefoot	28.15 ± 5.96	42.41 ± 9.69^a,b,c^	28.34 ± 6.68	26.89 ± 8.02	**0.012**	**0.003**	**0.009**
Midfoot	24.31 ± 7.7	33.69 ± 6.53^a,b,c^	22.48 ± 5.27	21.54 ± 5.9	**0.032**	**0.001**	**0.004**
Hindfoot	33.76 ± 10.35	45.92 ± 7.46^a,b,c^	33.95 ± 8.72	29.66 ± 6.92	**0.025**	**<0.001**	**0.014**

*P*_*LH*_, is the significance test between people with DPN and lower loading, and people with DPN and higher loading; *P*_*HH*_, is the significance test between people with DPN and higher loading, and people without DPN and higher loading; *P*_*HL*_, is the significance test between people with DPN and higher loading, and people without DPN and lower loading.

a Indicates a significant difference between people with DPN and lower loading, and people with DPN and higher loading (*p*< 0.05).

b Indicates a significant difference between people with DPN and higher loading, and people without DPN and higher loading (*p*< 0.05).

c Indicates a significant difference between people with DPN and higher loading, and people without DPN and lower loading (*p*< 0.05). P value in bold text indicate the significant difference. DPN: people with diabetic peripheral neuropathy; Non-DPN: people without diabetic peripheral neuropathy.

There was no significant interaction between the peripheral neuropathy and plantar loading on plantar pressure (*p* > 0.05). Peripheral neuropathy and plantar loading caused a significant main effect on plantar pressure, respectively (Table 2). The static PPP of participants in the DPN group was higher than Non-DPN group, with a significant difference over the whole foot, toes, forefoot, and hindfoot region (*p* < 0.05). The PTI of participants in the DPN group was significantly higher than Non-DPN group over the toes region (*p* < 0.05). In comparison to participants in lower loading group, people with higher loading showed significantly higher static PPP, dynamic PPP and PTI over the midfoot region and lower dynamic PPP over the toes region (*p* < 0.05).

In addition, people with callus over the forefoot region had significantly greater values of tissue hardness compared people without callus in the DPN group (50.94 ± 7.37 vs. 31.87 ± 6.19 Shore, *p* < 0.05). Their plantar pressure also higher than people without callus (static PPP: 232.18 ± 39.33 *vs.* 149.99 ± 36.69 kPa, *p* < 0.05, dynamic PPP: 764.69 ± 123.29 *vs*. 569.73 ± 179.61 kPa, *p* < 0.05, dynamic PTI: 124.25 ± 48.15 *vs*. 96.32 ± 27.66 kPa*s, *p* = 0.188).

## Discussion

This study investigated the relationship between plantar tissue hardness and plantar pressure in people with and without DPN, and preliminarily explored the influence of plantar loading associated with daily activities on plantar pressure and tissue hardness. The results showed significant correlations between tissue hardness and static PPP, and between tissue hardness and dynamic PTI in the forefoot region in people with DPN. Peripheral neuropathy and plantar loading caused a significant interaction effect on tissue hardness, but not plantar pressure. The plantar pressure distribution was independently associated with peripheral neuropathy and plantar loading. In comparison to people without DPN, significant differences in tissue hardness were only found in people with DPN and higher loading.

The results of this study showed that plantar tissue hardness of people with DPN was significantly correlated to static PPP and dynamic PTI over the forefoot region. This suggested the potential relationship between increased plantar tissue hardness and high plantar pressure. This study is an important supplement to Jan et al.'s study (Jan et al., 2013) that did not pay attention to the static plantar pressure. Static plantar pressure, reflecting the contact force of the foot with the ground during standing, is as important as dynamic plantar pressure in assessing the risk of DFUs (Duckworth et al., 1985; Patry et al., 2013). Thus, both static plantar pressure (during standing) and dynamic plantar pressure (during walking) were measured in this study. However, except the forefoot region, no significant correlation was observed between tissue hardness and plantar pressure in other plantar regions, which may be due to the fact that the forefoot is the main load-bearing area during daily activities. There was no significant correlation in the heel region may be related to the imbalanced plantar pressure distribution (Caselli et al., 2002; Kernozek et al., 2013; Al-Angari et al., 2017), which may lead to change of plantar load-bearing position. Besides, the different correlation trend between plantar pressure and tissue hardness in different plantar regions may be related to different injury thresholds, which should be explored in future studies. It should also be mentioned that no significant correlation between plantar pressure and tissue hardness was observed in people without DPN. This may be due to their relatively normal plantar pressure distribution and postural control during walking. The changes of the soft tissue biomechanical properties in diabetic people without DPN may be more influenced by the accumulation of advanced glycation end-products. Therefore, foot deformities, postural control and balance function may be considered in future studies. In this study, a significant correlation between increased plantar tissue hardness and plantar pressure could contribute to understand the changes of soft tissue biomechanical properties in people with DPN.

The findings of this study also found that increased plantar tissue hardness associated with peripheral neuropathy was affected by plantar loading level. People with DPN and higher loading had higher tissue hardness compared with people with DPN and lower loading in the forefoot, midfoot and hindfoot regions. It indicated a low shock-absorbing capacity to distribute mechanical stress during daily activities, especially weight-bearing activities (e.g. walking) in people with DPN. Excessive and repetitive plantar pressure loading may aggravate the stiffness of plantar soft tissue due to their weak ability to evenly distribute abnormal plantar pressure (Chatzistergos et al., 2014) during daily activities. Klaesner et al. demonstrated that the plantar soft tissue of people with DPN over the metatarsal heads was stiffer than healthy people using an indentor system (Klaesner et al., 2002). Several studies have also reported consistent findings using ultrasound palpation system (Sun et al., 2011). However, none of these studies involved diabetic people without DPN, which makes it difficult to determine the changes in biomechanical properties of plantar soft tissue associated with pure neuropathy. Only one study reported a significant difference in tissue hardness between diabetic people with and without DPN (Periyasamy et al., 2012b), which was consistent with the results in this study. Increased plantar tissue hardness in people with DPN and higher loading indicated a warning that excessive plantar loading during weight-bearing activities may increase the burden of fragile soft tissue caused by peripheral neuropathy (Sun et al., 2011) and make a negative effect on plantar soft tissue.

However, no significant difference was observed in plantar tissue hardness between people without DPN and lower loading and people without DPN and higher loading, which indicated the specificity and importance of the safe threshold for plantar loading during daily activities. The Physical Stress Theory (PST) proposed by Muller and his group assumes a window of “increased tolerance” between function maintenance threshold and injury threshold of plantar soft tissue. Physical stress within this window may be beneficial to enhance the adaptability of plantar soft tissue to external stress stimulus (Mueller and Maluf, 2002; Kluding et al., 2017) for people with DPN. The most important and challenging thing, however, is how to determine this safe threshold. In addition, Chao et al. showed that the stiffness of plantar soft tissue was increased in all diabetic people (diabetics with foot ulceration group, diabetics with neuropathy group, and pure diabetics group) compared healthy people, but no significant difference was reported between people with neuropathy and pure diabetics (Chao et al., 2011). This may be due to a lack of consideration of various plantar loading levels on soft tissue. In this study, no significant differences in tissue hardness were found between people with DPN and lower loading and people without DPN and lower loading. It indicated that appropriate plantar loading (e.g. performing weight-bearing physical activities) may be useful to improve the soft tissue biomechanical properties in people with DPN (Otterman et al., 2011; Mueller et al., 2013). Otterman et al. demonstrated the benefits of a 12-weeks exercise programme, consisting 30 min of aerobic exercise (e.g. cycling, walking, etc.) per day for people with DPN (Otterman et al., 2011). Mueller et al.'s study conducted a 12-weeks exercise programme for 1-h exercise sessions with 3 times per week (Mueller et al., 2013), and demonstrated the benefits of weight-bearing exercise in ambulatory function. Future studies may need to clarify the effects of different levels of plantar loading on plantar soft tissue, in order to seek the safety thresholds in people with and without DPN and guide physicians to develop exercise program for people with diabetes.

In addition, the static PPP of people with DPN were significantly higher than people without DPN, which was consistent with previous studies (Sacco et al., 2014; Halawa et al., 2018). However, no significant difference in dynamic PPP between people with and without DPN was observed in this study. Such differences may be influenced by different patient characteristics such as severity stages of diabetic peripheral neuropathy (Sacco et al., 2014) and skin health characteristics (i.e. callus presence). Because Sacco et al. found that plantar pressure gradually increased with the aggravation of neuropathy (Sacco et al., 2014). People with higher loading had higher plantar pressure in the midfoot region and lower plantar pressure in the toes region, suggesting changes of plantar pressure distribution under repeated mechanical stress stimulus. Therefore, the influence of such changes of plantar pressure distribution on diabetic foot ulcers still needs to be further studied.

In people with DPN, the forefoot region with callus had higher peak plantar pressure compared with people without callus. Studies suggested high shear stress near callus could cause abnormal peak plantar pressure and plantar pressure gradient in plantar soft tissues (Chao et al., 2011). Plantar soft tissue in callus area has impaired shock-absorbing function, which may result in tissue inflammation, skin breakdown and ulceration (Sun et al., 2011). Therefore, callus presence should be noticed immediately for people with DPN. It is necessary to ensure proper footwear and perform weight-bearing physical activities selectively.

The power analysis was performed to validate the statistical results of comparisons. There are large different effects for the comparisons of plantar tissue hardness in the whole foot and forefoot region between people with DPN and higher loading and people without DPN and higher loading (whole foot: 97.91%, forefoot region: 92.49%). In addition, the power of the difference in plantar tissue hardness in the whole foot and forefoot region between people with DPN and lower loading and people with DPN and higher loading was 80.77 and 88.55%, respectively. This may suggest an important influence of plantar loading level (i.e. weight-bearing physical activity duration) on the biomechanical properties of plantar soft tissue in people with DPN.

This study has some limitations. Firstly, plantar loading caused by exercise may result in different plantar pressures between people with and without DPN. It is necessary to examine the relationship between plantar loading and plantar pressure in a larger cohort of participants with DPN. Besides, plantar loading patterns were only divided into two levels based on the duration of daily weight-bearing activities, due to the limited sample size. More groups of plantar loading levels should be explored in the future. Secondly, this study explored the influence of plantar loading caused by exercise on plantar tissue hardness and plantar pressure in people with DPN. Future research should perform longitudinal studies to further explore the changes in the soft tissue biomechanical properties under long-term physical activities. Thirdly, walking was the main type of weight-bearing activities among the participants enrolled in this study. Other types of physical activities should be considered in future studies. Fourthly, the shore durometer has limitations in characterizing nonlinear viscoelastic behavior and tissue thickness of soft tissue. Ultrasound imaging may provide additional information on the biomechanical properties of plantar soft tissue (e.g. skin thickness). In addition, body weight and duration of diabetes may affect the results observed in our study (Abouaesha et al., 2001; Pirozzi et al., 2014; Jeong et al., 2021). Thus, our finding may not be generalized to people with DM who have different durations of diabetes and BMI. The influence of other covariates on plantar pressure and tissue hardness should be investigated in the future, such as body weight and skin quality on different regions of foot. Fifthly, the plantar surface was divided into four regions in order to explore the potential relationship between tissue hardness and plantar pressure in the whole plantar region in people with diabetes. Subdivision of plantar regions (e.g. the five metatarsal regions of the forefoot, big toe and little toes) should be considered in future studies.

## Conclusion

In conclusion, this study found that the plantar tissue hardness was correlated to plantar pressure in people with DPN. Peripheral neuropathy and plantar loading patterns associated with various physical activities are important factors affecting the biomechanical properties of plantar soft tissue. The findings of this study contribute to further understand the relationship between increased plantar tissue hardness and high plantar pressure in people with diabetic peripheral neuropathy.

## Data Availability

The original contributions presented in the study are included in the article/Supplementary Material, further inquiries can be directed to the corresponding authors.
